# Evaluating online health information on PIFP: A cross-platform analysis of content quality and readability

**DOI:** 10.1038/s41598-025-24426-5

**Published:** 2025-11-18

**Authors:** Dina Taimeh, Alaa Atef, Hamza Abu Shawish, Mohammad Atrash, Mohammad Qaralleh, Yara Flaifl

**Affiliations:** 1 Department of Oral and Maxillofacial Surgery, Oral Medicine and Periodontology, School of Dentistry, The University of Jordan, Amman, Jordan; 2 The University of Jordan Hospital, Amman, Jordan; 3 School of Dentistry, University of Jordan, Queen Rania St., Amman, 11942 Jordan

**Keywords:** Chronic pain, Facial pain, Online information, Quality, Readability, Health care, Mathematics and computing, Medical research

## Abstract

Persistent Idiopathic Facial Pain (PIFP), also known as atypical facial pain, is a poorly understood condition, often leading patients to seek information online. The accuracy, readability, and usability of such information are critical for informed decision-making. This study aimed to assess and compare the readability, quality, and usability of online content about PIFP retrieved from both conventional web searches and generative AI platforms. A cross-sectional comparative analysis was conducted on January 26th 2025 using three search. Google, ChatGPT, and Gemini. Google was searched using the terms “Atypical Facial Pain” and “Persistent Idiopathic Facial Pain,” and the first 100 results for each term were screened. ChatGPT and Gemini were prompted to generate the ten most frequently asked questions about PIFP. Selected content was evaluated using the Patient Education Materials Assessment Tool (PEMAT) for understandability and actionability, the Journal of American Medical Association (JAMA) benchmarks for content quality, and two readability measures: Flesch Reading Ease and the Simple Measure of Gobbledygook (SMOG) formulae. A total of 43 websites were included. Only 44.1% of websites met at least one JAMA benchmark, while 55.9% met none. AI-generated content failed to meet any JAMA criteria. However, AI content was significantly more understandable (mean score: 83.31%) than traditional websites (mean: 64.96%, p < 0.001). In contrast, websites had higher actionability scores (25.25%) compared to AI-generated content (11%, p < 0.001). All sources were rated as “difficult” to read. Online information regarding PIFP is often difficult to read and generally lacks both quality indicators and actionable guidance. While AI-generated content is more understandable, it lacks practical advice. These findings underscore the need for improved online patient education materials that are both high in quality and easy to understand, especially for complex pain conditions like PIFP. Health professionals and digital content developers should collaborate to ensure that online resources meet standards for clarity, reliability, and usefulness.

**Supplementary Information:**

The online version contains supplementary material available at 10.1038/s41598-025-24426-5.

## Introduction

Persistent idiopathic facial pain (PIFP), previously known as ‘atypical facial pain (AFP)’, is characterised by persistent facial and/or oral pain, with varying presentations but recurring daily for more than 2 h a day over more than 3 months, in the absence of clinical neurological deficits^1^. According to the International Headache Society, this diagnosis is given when, among other criteria, a normal clinical neurological examination is noted and dental causes have been excluded^1^. Given that it is an idiopathic pain condition in which no relevant abnormalities are found, the diagnosis is made by excluding other facial pain conditions^2^.

There is little high-quality evidence regarding PIFP, and the aetiology remains unclear. However, as with other chronic pain conditions, central sensitisation is suggested^3^. Additionally, many patients diagnosed with PIFP report a history of mild trauma or dental work, albeit disproportionate to the reported pain level^4^. As such, the current literature places PIFP at one end of a spectrum with posttraumatic trigeminal neuropathy (PTTN) at the other end^1,4^. Psychological comorbidities are also identified, possibly playing a role both as risk factors and as consequences of living with chronic pain conditions. Increased scores for anxiety and depression are common, especially in patients reporting higher pain intensity^4,5^.

Patients with chronic pain are increasingly using the web to find information about their condition. As the internet is becoming an ever-growing part of people’s lives, individuals are becoming more reliant and comfortable in using it as a source of medical information^6^. In fact, some reports estimate that up to 80% of patients use the internet at one point while seeking health-related knowledge^7^. It is also estimated that patients with chronic illnesses are more likely to source information related to their condition on the internet^8,9^. Artificial intelligence (AI) is also rapidly gaining popularity among consumers. There is no single or simple definition for AI, however, it refers to computer systems that can perform complex tasks normally done by human-reasoning, decision making and creating^10^.

Many examples in the current literature describe the role of the internet for patients with chronic pain. For example, patients may derive support from each other by sharing their experiences with each other through forums and support groups^11^. Patients may also use online information for practical advice, self-management techniques, reassurance and comparisons of different treatment modalities. This highlights the ever-growing influence of the internet, which cannot be understated. Indeed, some authors report that patients may search the web as soon as symptoms appear or are aware of a label to their condition^11^. Therefore, it is imperative that healthcare providers communicate reliable, trustworthy and easy-to-read information. To this end, various organisations have made recommendations regarding the development of health-related material. For example, the American Medical Association (AMA) released a manual for clinicians as part of an educational programme about health literacy, whereby it emphasised the importance of patient education material. Among the recommendations outlined was the suggestion that text should be constructed at or below 6th grade level^12^. Consequently, patients who are better informed about the diagnosis, risk factors, consequences, and available treatments, demonstrate enhanced compliance with medication across a wide range of conditions and improved health behaviours^13^.

To the best of our knowledge, no previous studies have evaluated the quality of online information regarding PIFP or AFP. Thus, the aims of the present study were to assess the readability and quality of online information regarding PIFP and AFP and compare the results generated by three search methods (Google.com, ChatGPT and Gemini).

## Materials and methods

This cross-sectional study examined the generated online results at one point in time. It did not involve human or animal subjects. As such, it was exempt from institutional ethical review, and from obtaining informed consent from subjects.

### Website identification and artificial intelligence response

An online search was conducted on the 26th of January2025 on Google.com using the terms “Persistent Idiopathic Facial Pain” and “Atypical Facial Pain”. The first 100 websites for each term were documented and screened for inclusion. The search was carried out on both a laptop and a smart mobile phone. Hence, 400 websites were screened. The websites eligible for inclusion were websites in the English language and contained information related to PIFP or AFP. Websites were excluded if they primarily targeted healthcare professionals (e.g., journal articles and book chapters), if they contained irrelevant information, extremely short material, or if they required registration to gain access^14,15^. Owing to the dynamic nature of the internet, the links for all the websites (both on a PC and a mobile phone) were documented in one sitting on an Excel sheet (by HAS and MA) and were shared with the research team.

Another separate online search was conducted using ChatGPT and Gemini chatbots in a ‘signed out’ mode. The first step involved determining the most frequently used questions regarding PIFP and AFP in AI-based models. For this purpose, the following questions were asked separately in English to the chatbots: “What are the 10 most frequently asked questions about persistent idiopathic facial pain/atypical facial pain?”. These 40 queries (20 regarding PIFP via the two AI-based models and 20 regarding AFP via the same two models) were asked individually, and the responses were recorded on the same day for appraisal of quality and readability. See the supplementary material for the individual questions that resulted from the search (S1).

### Website characterisation

After screening and filtering the websites against the inclusion and exclusion criteria, they were categorised according to their source of upload (commercial entities, non-profit organisations, universities, and governmental sources). The following information was also recorded: the country of upload, the content specificity (exclusively or partly related to the topic), and the use of media (images, videos and audio content)^14,15^.

### Quality and readability of information

Two tools were used to assess the quality of the online material: the Patient Education Materials Assessment Tool for printed material (PEMAT-P)^16^ and the Journal of the American Medical Association (JAMA) benchmarks^17^. The PEMAT is composed of two domains: “understandability” and “actionability”. Patient education material is considered “understandable” if readers of variable literacy levels can process and explain key messages. It is assessed by rating 15 items as 1, 0 or N/A (corresponding to agree, disagree or not applicable, respectively). The material is considered “actionable” if readers can also identify what they can do on the basis of the information provided. This domain consists of seven items rated as 1 or 2, with two of the items also including the N/A option. Eventually, a score is given for each domain separately in the form of a percentage by summing the total points, dividing the sum by the total possible points, and multiplying the result by 100. According to the PEMAT guidance, the higher the score is, the more understandable and actionable the material is. A score above 70% is generally considered an acceptable score for both understandability and actionability^16,18^. The PEMAT manual was consulted regularly during the appraisal process^19^.

The JAMA benchmarks were used to analyse the accountability of the material^20^. The instrument checks for the inclusion of four criteria: authorship, attributions, disclosure (if there is any conflict of interest or ownership), and currency (date of upload)^17^.

The readability of the material was evaluated via two tools: the Flesch Reading Ease Score (FRES) and the Simple Measure of Gobbledygook (SMOG) readability formula. The scores were calculated by copying the text from the websites into an online calculator (available on http://www.readabilityformulas.com/). The text is then automatically analysed to generate the scores based on both formulae. The first tool estimates the ease of comprehension of a piece of text and the level of education needed to appreciate it^21^. The scores range from 0 to 100, with higher scores reflecting easier text readability. Scores ranging from 0 to 29 are considered “very difficult to read”, scores ranging from 30 to 49 are “difficult”, scores ranging from 50 to 59 are “fairly difficult”, scores ranging from 60 to 69 are “standard”, scores ranging from 70 to 79 are “fairly easy”, scores ranging from 80 to 89 are “easy”, and scores ranging from 90 to 100 are “very easy”. Scores ranging from 80 to 89 are considered at conversational level for consumers, and correspond to 6th grade level.

The second tool estimates the years of education a person needs to understand written material. The higher the SMOG score is, the more difficult the material is to read^22^. It measures the number of years of education the average person needs to understand a text, with a score of 6 meeting the readability recommendation previously mentioned^23^.

Two reviewers assessed the included websites separately (YF and AA). Five websites were initially evaluated jointly by the reviewers for calibration purposes. In cases of discrepancy in the results, a third reviewer (MQ) was consulted. The agreement between reviewers 1 and 2 was calculated via the kappa coefficient and was found to be 0.93.

Similar to the output from the AI models, two reviewers evaluated the material separately (HAS and MA) after assessing the five generated text materials jointly for calibration purposes. A third reviewer was consulted in case of discrepancy in the results (MQ). The agreement between reviewers 1 and 2 was also calculated via kappa and was found to be 0.933.

### Statistical analysis

The data was summarised via descriptive statistics as the first step. The Shapiro‒Wilk test was also conducted to explore the normality of the continuous data prior to any further analysis. Student’s t-test was used for normally distributed data, and the Mann‒Whitney test was used for non-normally distributed data. All tests were two-tailed, and p values < 0.05 were considered statistically significant. The statistical analysis was performed via the IBM SPSS statistical package for Windows, version 27.0 (SPSS Inc., Chicago, IL).

## Results

### Website identification and characteristics

**Fig. 1:**
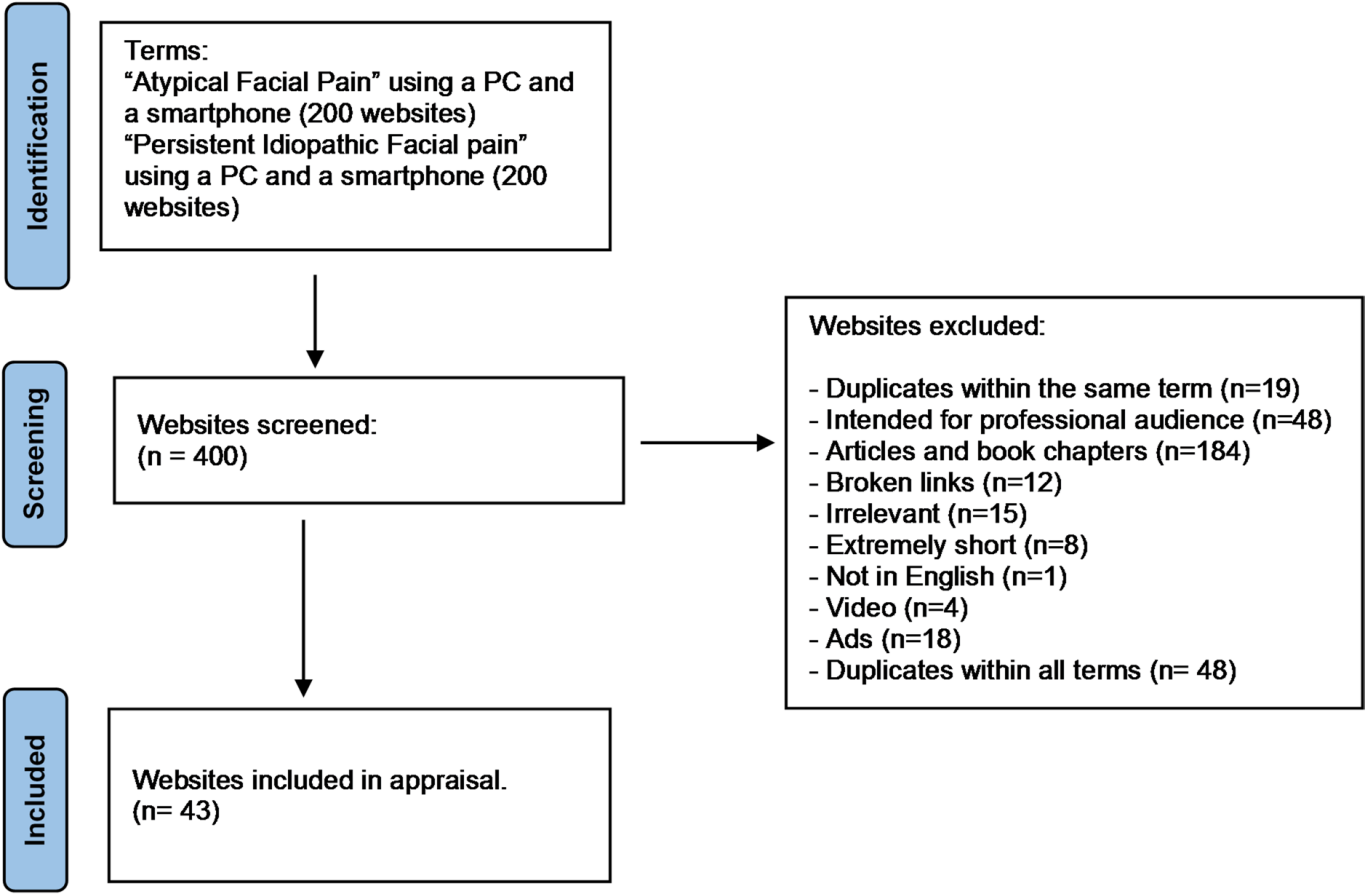
Flow chart of website identification and inclusion.

After the search was conducted on Google.com via the terms “Persistent Idiopathic Facial Pain” and “Atypical Facial Pain”, the first 100 websites for each term were screened. After applying the exclusion criteria, 11 websites were included for the term “Persistent Idiopathic Facial Pain”, and 34 were included for the term “Atypical Facial Pain”, for a total of 45 websites. Using a mobile phone search, 12 websites were included for the term “Persistent Idiopathic Facial Pain”, and 34 were included for the term “Atypical Facial Pain”, totalling 46. The total number of websites that were appraised for both terms via the two methods of searching after removing duplicates was 43 websites. Figure 1 describes the identification and exclusion process. When examining the terms “AFP” and “PIFP” separately after removing the duplicates within the same term (including both the pc and mobile results), 40 websites were included for “AFP”, and 13 were included for “PIFP”. See the supplementary material (S1) for the characteristics of the included websites.

A total of 74.4% of the websites originated from the USA (*n* = 32), followed by the UK and India. A total of 76.7% were commercially affiliated (*n* = 33). The majority of the included websites were exclusively related to AFP or PIFP (*n* = 36), and 55.8% displayed media content (*n* = 24). Table 1 lists the website characteristics in detail.

**Table 1:** Details of the website characteristics.

	Category	Percent (*n*)
Affiliation	Commercial bodies	76.7% (*n* = 33)
	Universities	2.3% (*n* = 1)
	Government sources	2.3% (*n* = 1)
	Nonprofit organisations	18.6% (*n* = 8)
Specialisation	Exclusively related to AFP/PIFP	83.7% (*n* = 36)
	Partly related to AFP/PIFP	16.3% (*n* = 7)
Content type	Medical content	95.3% (*n* = 41)
	Human interest stories	0% (*n* = 0)
	Questions and answers	4.7% (*n* = 2)
Media	Contains media	55.8% (*n* = 24)
	No media (image/video/audio)	44.2% (*n* = 19)
Country of origin	USA UK India Netherlands Singapore No available data	74.4% (*n* = 32) 11.6% (*n* = 5) 7% (*n* = 3) 2.3% (*n* = 1) 2.3% (*n* = 1) 2.3% (*n* = 1)

### Quality of information

Only one website displayed all four JAMA benchmarks (affiliated with a non-profit organisation). A total of 44.1% of the websites displayed at least one benchmark (*n* = 19), and 55.9% of the websites displayed no benchmark at all (*n* = 24). The JAMA benchmark most frequently displayed was authorship (*n* = 17), and the benchmark least frequently displayed was disclosure (*n* = 2). See Table 2 for more details. Interestingly, the AI-generated material did not display any of the JAMA benchmarks at the time of this study.

**Table 2:** Percentage and frequency of websites displaying the JAMA benchmarks.

Number of JAMA benchmarks	Percent (*n*)
0 benchmark	55.9% (*n* = 24)
1 benchmark	11.6% (*n* = 5)
2 benchmarks	18.6% (*n* = 8)
3 benchmarks	11.6% (*n* = 5)
4 benchmarks	2.3% (*n* = 1)

The mean PEMAT score for understandability for all the included websites was 64.96% (± 14.2 SD). The minimum and maximum scores were 33.3% and 91.67%, respectively, which were both achieved by websites affiliated with non-profit organisations. When both terms were examined separately, the mean understandability score for AFP was 64.91% (± 14.2 SD), whereas that for PIFP was 64.50% (± 16.88 SD). However, there was no statistically significant difference between the two means according to the t test (p value = 0.932).

The mean PEMAT score for actionability was 25.25% (± 23.06 SD). The minimum and maximum values ranged from 0.0% to 85.71%. The websites associated with zero actionability numbered eight in total; three were affiliated with non-profit organisations, and five were commercially affiliated. Moreover, the maximum score was achieved by a website affiliated with a non-profit organisation. The mean actionability score for AFP was lower than that for PIFP: 23.0% (± 19.5 SD), as opposed to 35.82% (± 31.44 SD). No significant difference was noted between the two categories when they were compared via the Mann‒Whitney test (p value = 0.224).

With respect to the PEMAT scores for the AI-generated material, a mean understandability score of 83.31% (± 6.08 SD) was achieved, which was significantly higher than the understandability scores for the websites from Google.com (p value < 0.001). On the other hand, the mean PEMAT scores for actionability were much lower for the AI-generated material, with a mean of 11% (± 18.08 SD), than for the Google output (p value < 0.001).

Table 3 details the means and associated p values when comparing the PEMAT scores for the Google results and AI-generated material. The understandability of the Google results was significantly lower than the AI-generated material for total understandability, understandability for the terms AFP and for PIFP (p value of < 0.001 for all three). As for the actionability, Google results had statistically higher scores when compared to AI-generated material for total actionability and actionability for the terms AFP and PIFP (p values of 0.001, 0.002, and 0.042 respectively).

**Table 3:** Means and p values of the PEMAT scores comparing Google results and AI-generated material.

	Total-U	*P* value	AFP- U	*P* value	PIFP- U	*P* value
Google	64.96%	†<0.001*	64.91%	†<0.001*	64.50%	†<0.001*
AI material	83.31%		83.83%		82.78%	
	Total- A	*P* value	AFP- A	*P* value	PIFP - A	*P* value
Google	25.25%	‡0.001*	23.0%	‡0.002*	35.82%	‡0.042*
AI material	11%		8%		14%	

U: PEMAT understandability. Total U: understandability when AFP and PIFP are combined. AFP: atypical facial pain. PIFP: persistent idiopathic facial pain. A: PEMAT actionability. †: t test. ‡: Mann‒Whitney test. *: P value < 0.05 indicating statistical significance.

### Readability of information

The mean Flesch Reading Ease score for the included websites was 43.88 (± 11.08 SD), meaning that it was “difficult to read”. The scores ranged from 21 (very difficult to read) to 73 (fairly easy to read). When the scores for AFP and PIFP were compared separately, the readability score for AFP was slightly lower than that for PIFP; however, they were both “difficult to read” (43.66 and 45.09, respectively). For the AI-generated material, the mean Flesch readability score was 39.55 (± 8.68 SD). The ChatGPT and Gemini outputs for AFP were also higher than the output for PIFP; however, all mentioned values fell within the “difficult-to-read” category. See Table 4 for detailed information regarding the mean of each category.

**Table 4:** Means of the readability scores as measured by the FRES and SMOG formulas.

	FRES mean (± SD)	SMOG mean (± SD)
AFP		
Google	43.6 (± 11.07 SD)	10.35 (± 1.84 SD)
ChatGPT	40.6 (± 8.98 SD)	10.806 (± 1.45 SD)
Gemini	41.2 (± 12.47 SD)	10.932 (± 1.76 SD)
PIFP		
Google	45.09 (± 11.34 SD)	10.10 (± 1.74 SD)
ChatGPT	39.1 (± 5.56 SD)	11.017 (± 1.08 SD)
Gemini	37.3 (± 7.04 SD)	11.387 (± 0.69 SD)

FRES: Flesch reading ease score. SMOG: Simple measure of gobbledygook. SD: standard deviation.

The mean SMOG score for all the included websites was 10.32 (± 1.79 SD), corresponding to the Grade 10 level. Similar results were noted when the included websites for AFP and PIFP were compared separately (10.35 and 10.10, respectively), both of which also corresponded to Grade 10 level reading difficulty. The SMOG readability score for the AI-generated material was 11.04 (± 1.27 SD), meaning that Grade 11 reading was needed. Similarly, the reading levels for AFP (using both ChatGPT and Gemini) and PIFP corresponded to Grade 11 levels. Table 4 shows the mean for each category.

## Discussion

Over the past few decades, an extraordinary shift has occurred in the accessibility of knowledge. In 1993, the Worldwide Web was launched into the public domain, and since then, it has altered society in many ways. While in the traditional sense, medical knowledge has been almost confined to textbooks and medical journals, it is now available easily and quickly for most people. The National Center for Health Statistics reported that from July–December 2022, 58.5% of adults in the U.S. used the internet to look for health or medical information^24^. As a consequence of this surge in the availability of medical data, a new term was introduced into the literature: the “e-patient”^25,26^. E-patients tend to gather information about their health and actively engage with their doctors during visits by asking relevant and critical questions^27^. Thus, they are more informed about their health and are potentially better able to make decisions and be in a partnership with the clinician^25^. On the other hand, the information available on the internet might be poorly moderated and hence risk the spread of false, or even harmful, information^28^. In fact, a finding published in 2009 by the Pew Research Center in the U.S. depicted that 3% of e-patients reported that they or someone they knew had been harmed by following medical advice or health information found on the internet^29^.

The recent introduction of AI also has a potential role in revolutionising healthcare. It is a rapidly evolving technology that is increasingly gaining popularity. Chatbots such as ChatGPT, Gemini and DeepSeek are systems designed to process and understand human queries by retrieving data from internet databases and generating human language via their advanced natural language processing (NLP) model^30,31^. Several applications are emerging in healthcare, such as assisting in diagnostics and treatment, assisting in population health management and providing virtual healthcare assistance, such as AI mental health support. One useful application is patient education and mitigating healthcare provider burnout^32^. Examples include the promotion of physical activity^33^, diet recommendations and weight loss^34^. AI-based chatbots can also provide personalised information and guidance to patients and help patients understand their medical diagnosis and treatment options^26^. It also has the potential to transform patient education and communication by enhancing the delivery of information and improving patient engagement. Interestingly, the search output from Google.com began incorporating an AI overview at the beginning of the search after the study was conducted. For example, the output for “Atypical facial pain” provided several paragraphs explaining the “Key characteristics” of the AFP. However, a few points noted by the authors were the use of medical jargon when explaining the condition, such as “neurological deficits”, “multidisciplinary approach” and “diagnosis of exclusion”. Another observation was regarding the reference list displayed in association with the AI overview. While it contained some reliable sources, such as journal articles, some of the other references were private clinics and online encyclopaedias written and edited by volunteers, not necessarily medical personnel. A footnote was also incorporated to highlight that this information was for informational purposes only and to seek medical advice for diagnosis. As such, this technology undoubtedly comes with challenges and limitations, such as ensuring the reliability and accuracy of the material and securing the patients’ confidentiality and data^35^. The Pew Research Center also reported that despite the enthusiasm of AI experts, a large proportion of US adults view AI in a negative light and worry about too little regulation^36^. Hence, health literacy is relevant in this context, meaning that the individual is able to access, understand, appraise the information and use it in a way that promotes and maintains good health and well-being^37^. Therefore, the World Health Organisation advises that all information providers, including governments and health services, should provide trustworthy information that is understandable and actionable for all people^37^. However, there are still concerns regarding health literacy, even in developed countries. For example, estimates regarding health literacy in the United States of America indicate that approximately 36% of adults have basic or below-basic health literacy^38^. In the UK, 7.1 million adults read and write at or below the level of a nine-year-old child, and 43% of adults do not understand written health information^39^. This highlights the need for high-quality, accurate and easy-to-read information from reliable sources that can be shared with patients to educate them about various health conditions. Patients who are better informed and educated are suggested to have better adherence to treatment regimens and are more likely to achieve better health outcomes^26,40^.

Chronic pain is expected to cause certain challenges for patients, such as the effects it may have on social and professional aspects of life^41,42^. This may be more common in chronic pain conditions with no tangible evidence of pathology, such as fibromyalgia and myogenous temporomandibular disorders. In fact, multiple reports highlight the struggle of chronic pain patients to validate their pain experience and to be believed^43–45^. These feelings of doubt may permeate their experiences with family members, work colleagues and health care providers^46^. Hence, it is not unreasonable to assume that patients may seek information online to validate their experiences and feelings.

The present study aimed to assess the readability and quality of the information available online regarding PIFP and AFP. Both terms describe the same idiopathic chronic facial pain condition, where the diagnosis is reached by excluding all other facial pain conditions that share similar symptoms first. The websites that resulted from the search were extracted and documented via an Excel sheet in one sitting due to the instability of the online results. The first 100 websites for each term were screened, as evidence suggests that only a minority of internet users browse beyond the first 10 pages when looking for a piece of information^47^. More of the websites that were screened were included for appraisal when the term AFP was used than when the term PIFP was used, suggesting that the former is more commonly used in patient-targeted material. This may be due to the shorter term and easier pronunciation. PIFP is also possibly more frequently used in material targeted for medical personnel, as evidenced by the number of excluded websites targeted for professional audiences, such as journal articles and book chapters.

The majority of the websites were commercially affiliated, for example, private dental or medical practices. This finding indicates that commercial bodies may devote more effort to reaching wider audiences by promoting their websites. This also highlights the need for more patient-targeted material from governmental or academic sources, where the reliability and accuracy of information can be more readily presumed.

When examining the quality of information, where it is presumed to be reliable if at least three out of the four JAMA benchmarks were evident^17^, shortcomings were noted. The majority of the included websites failed to achieve this goal. Interestingly, these findings are reported in other studies examining the online content of various health-related topics, such as oral leukoplakia^48^ and oral health in patients with autism^15^. Studies examining the online content of material about chronic pain also reported shortcomings in regards to quality and credibility^49,50^ This is a cause for concern, as the quality of online information circulated to patients cannot be guaranteed. Perhaps more so in chronic pain conditions, as receiving a diagnosis may be a challenge, and healthcare experience may be less than ideal^46,51,52^. This group of patients highlighted the challenges of obtaining a ‘label’ for their pain condition by healthcare providers. Hence, turning to alternative sources such as the internet is not implausible. Interestingly, the AI-generated material also lacked all four JAMA benchmarks at the time of the search, meaning that authorship, attribution, disclosure and currency of the data cannot be inspected. However, AI technology is rapidly evolving, and after running the search for the purpose of this study, the search engines started displaying a reference list at the end of the query unprompted.

The mean PEMAT scores for understandability were less than the 70% cut-off point when looking at the included websites from Google.com. This is also true for both terms (AFP and PIFP). Patient educational material is understandable when readers with varying levels of health literacy are able to process and explain key messages^19^, which, unfortunately, is not the case for the examined websites. AI-generated material, on the other hand, had significantly better understandability but much lower actionability than the material extracted from the websites. This means that the readers cannot identify what they can do on the basis of the information presented^19^. A footnote was inserted, however, at the end of the presented material, with the goal of seeking a healthcare provider if the readers were experiencing persistent facial pain. The actionability of online patient educational materials is often poor as reported in multiple studies in different fields^14,53^. Therefore, patients may find it difficult to take necessary action. However, actionability can be increased by applying simple measures; such as identifying at least one action to take, breaking down this action into manageable and explicit steps, addressing the reader directly, and using visual aids^19^.

While the PEMAT does not necessarily assess the accuracy or comprehensiveness of the material, it has been described in the literature as a means to measure the quality of a variety of patient educational materials^54^. The use of the PEMAT in conjunction with readability assessment tools to evaluate the suitability of the material to consumers is also recommended^19^. Two tools were used in this study to evaluate the readability of the material, as this tool is suggested to improve the reliability by approximately 0.20 when more than one formula is used^14,55^. The readability of the material extracted from Google and AI chatbots was considered difficult to read. These results do not meet the recommendations of several healthcare organisations, which recommend that the readability of patient information should not be higher than the 6th grade level^56^. This is particularly concerning as this trend was found in the perceived “popular” and even presumably reliable websites.

The Centers for Disease Control and Prevention (CDC) recommends reducing reading levels in patient-related documents by suggesting several techniques. Examples include reducing the number of words per sentence, using one- or two-syllable words when possible and using the active voice^57^. It also iterates that reading formulas are useful tools for providing a general idea of how difficult a document is to read. They do not, however, measure the level of comprehension, which is often two or more grades below the reading or education level^57^.

The benefits of high-quality, reliable and readable online information for patients with chronic pain cannot be understated. Previous studies have highlighted the importance of the internet for this group of patients; it is useful in finding information, feeling supported, maintaining relationships and affecting behaviours and experiences with healthcare^11^. However, several concerns remain, namely, the lack of robust regulations to manage the content, unequal access, and lack of trustworthy sources to represent disadvantaged populations. For example, English speakers may have access to many more web resources than any other language group does. Hence, some populations may find it difficult to locate material that reflects their experiences and resembles their own^11^.

Additionally, patients with chronic pain tend to seek validation for their pain as reported by numerous studies^58,59^. It could be through health care professionals, family members, and recently through internet sources^11^. This valuable source of information has a remarkable opportunity to shape the experiences of patients with chronic pain, especially idiopathic ones such as PIFP. It presents the opportunity for the patients to be better educated about their conditions and potentially changes their approach to treatment. This seems paramount as much evidence points to the importance of patient education in the management of chronic pain conditions, for example temporomandibular disorders and chronic musculoskeletal pain^60,61^,. Hence, having low-quality and difficult to read online information about these various health conditions where patients experience a higher risk of anxiety, depression and psychological comorbidities, might indeed be consequential.

## Strengths and limitations

This study was the first to examine the quality and readability of the online material regarding PIFP. Both terms used to describe this condition (AFP and PIFP) were used, to maximise the included material. Additionally, two reviewers appraised the extracted material independently in an effort to minimise subjectivity in assessment. Finally, multiple reliable and widely used tools were used to evaluate the website content and AI generated material.

Several limitations were associated with this study. First, the search methods used were of an ever-evolving nature. AI is a new and rapidly advancing technology, and while the results of this study reflect the nature of the material at the time, they may not, if the study was conducted at another time point. This, however, is a reflection of the dynamic nature of the internet and the rapidly changing content. Hence, studies with such methodologies are expected. Another limitation was the use of English-language content A single calculator (website) was also used to assess the readability of the material. Additionally, the present study appraised the readability and quality of the information, whereas accuracy was not assessed.

The online information regarding AFP and PIFP was generally deemed too difficult to read for lay people, as reflected by the readability scores. It had more or less acceptable understandability but low actionability. There is significant room for improvement in online patient-related material regarding atypical facial and/or persistent idiopathic facial pain. Healthcare organisations and governments have an essential role in improving patient educational material by focusing on accessibility and delivery methods. For example, they can provide reliable web-based resources to patients via social media, disseminate patient leaflets in healthcare centres, or personalise interactive apps based on diagnoses. Ensuring readable, understandable and culturally appropriate content is also important, especially for communities disadvantaged by language, internet access or location.

## Supplementary Information

Below is the link to the electronic supplementary material.

Supplementary Material 1

## Data Availability

Data is available from the corresponding author upon request.
